# Supplementary material for: Exploring Aromatic S-Thioformates as Photoinitiators
Source: Polymers (Basel). 2023 Mar 26;15(7):1647. doi: 10.3390/polym15071647 (PMC10097006; doi:10.3390/polym15071647)
Supplement: Supplementary file 1 [file polymers-15-01647-s001.zip › polymers-2203188-supplementary.pdf]

Exploring Aromatic *S*-Thioformates as Photoinitiators

Paul Rieger[1], Sabrina Pueschmann[2], Michael Haas[2], Max Schmallegger[3], Georg Gescheidt[3], Gema Guedes de la Cruz[1], Thomas Griesser[1]**

[1]Institute of Chemistry of Polymeric Materials, Montanuniveristät Leoben, Otto Glöckelstrasse 2, 8700 Leoben, Austria.

[2] Institute of Inorganic Chemistry, Graz University of Technology, Stremayrgasse 9, 8010 Graz, Austria.

[3] Institute of Physical and Theoretical Chemistry, Graz University of Technology, Stremayrgasse 9, 8010 Graz, Austria.

E-mail: thomas.griesser@unileoben.ac.at;
gema-del-carmen.guedes-de-la-cruz@unileoben.ac.at

Keywords: photochemistry, photoinitiator, thiol-ene

SUPORTING INFORMATION

All manipulations of oxygen- and moisture-sensitive materials were conducted under a nitrogen atmosphere in a flame dried Schlenk flask. ^{1}H-NMR spectra were recorded on a BRUKER 200 MHz spectrometer using CD_3CN and DMSO-d_6 as deuterated solvents. The ^{13}C-NMR spectrum was recorded on the same instrument at 50 MHz. Chemical shifts (δ) are expressed in ppm from downfield to upfield. For ^{1}H-NMR spectra, the multiplicity is indicated as singlet (s) and multiplet (m). For kinetic studies, two different types of NMR tubes were employed depending on the irradiation experiment: (1) a quartz NMR tube for UV irradiation with a monochromatic lamp (254 nm) and (2) a borosilicate glass NMR tube for visible irradiation with a monochromatic lamp (405 nm). The UV-Vis spectra were recorded on a Varian Cary 50 UV-Visible Spectrophotometer in the range of 300-800 nm at a scan rate of 600 nm/min, a data interval of 1.0 nm and an average time of 0.1 s, using a quartz cuvette with an optical path length of 10 mm. The FTIR spectra used to calculate the double bond (DB) conversation were measured using a PerkinElmer Spectrum One FT-IR spectrometer. Proving

the carbon monoxide (CO) formation was conducting by GC-MS, with a Thermo Scientific Trace 1300 Gas Chromatography and Thermo Scientific ISQ 7000 Single Quadrupole Mass Spectrometer, equipped with a fused silica capillary column (Optima-5-Accent-15 m x 0.250 mm x 0.25 µm film thickness), an automatic sampler, a split/splitless injector and a mass selective detector. The injector temperature and the GC-MS interface was kept at 250 °C. After injection of 1 µL sample, the column was heated from 40 to 260 °C with a heating rate of 12.94 °C/min. The helium carrier gas flow rate was set at 1.3 mL m^{-1}. The rheological measurements were performed on a MCR 102e system (Anton Paar). A plate-plate configuration (diameter of 25 mm) was used in oscillating mode with a frequency of 1 Hz and an amplitude (γ) of 1 %. The printing experiments were conducted using a Photon S 3D printer (Anycubic). Bottom layer exposure was 45 s, exposure of other layers was 30 s with a layer height of 20 microns, and with an average intensity of 2.14 mW cm^{-2}. Spin-trapping electron paramagnetic resonance (ST-EPR) was conducted in a Magnettech X-Band EPR spectrometer. 5,5-Dimethyl-1-pyrroline-N-oxide (DMPO) was employed as a trapping reagent and MeCN as solvent. The sample was irradiated with a Hamamatsu LC4 HgXe lamp (365 nm). The HPLC chromatograms were recorded on an Ultimate 3000 UHPLC system (Thermo Fisher) with a C18 reversed-phase analytical column (ACE, 2.1 x 100 mm, particle size 3 µm) at 35 °C using a mobile phase A (H_2O) and B (MeCN) at a flow rate of 0.5 mL min^{-1}. The following gradient was applied: linear increase from 30% solution B to 100% solution B in 15 min, hold at 100% solution B for 3 min. Melting point was determined using a microscope and controlled heating plate. All chemicals were obtained from Aldrich Chem. Co, Riedel-de Haën, TCI and Thiocure, and used as received.

1. Synthesis of *S*-phenyl thioformate (1b) and *S,S`*-(thio*bis*(4,1-phenylene))dimethanethioate (2b)

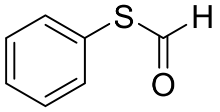

***S*-phenyl thioformate (1b)**: the synthesis of this compound was performed according to the literature.[1] A yield of 90% was obtained.

^{1}H-NMR (200 MHz, CD_3CN, δ): 10.24 (s, 1H), 7.52 (m, 5H) ppm.

[1] R. Haraguchi, S.-G. Tanazawa, N. Tokunaga, S.-I. Fukuzawa. Palladium-catalyzed formylation of arylzinc reagents with *S*-phenyl thioformate. *Organic Letters* **2017**, *19*, 1646-1649.

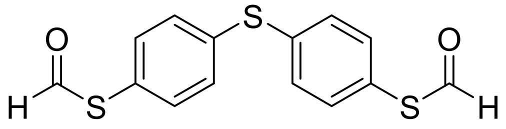

***S,S′*-(thio*bis*(4,1-phenylene)) dimethanethioate (2b)**: The reaction was performed in a 50 mL Schlenk flask (previously flame-dried under vacuum) equipped with a magnetic stirring bar. Under N_2 atmosphere, formic acid (3 mL, 80 mmol) and acetic anhydride (3.80 mL, 40 mmol) were added and stirred at 45 °C for 2 h. After cooling at rt and cover the flask with aluminium foil, 4,4′-thiol*bis*benzenethiol (2.50 g, 10 mmol) suspended in anhydrous THF (20 mL) and anhydride pyridine (0.03 mL, 0.37 mmol) were added and stirred at 30 °C under N_2 atmosphere for 24h. Subsequently, water was added (100 mL) and the mixture was extracted with EtOAc (2 x 100 mL). The combined organic layers were washed with brine, dried over sodium sulphate, and concentrated in vacuo. After checking the purity of the crude by ^{1}H-NMR, a second extraction was required; this time extracting only once with EtOAc. The purity of the product was corroborated by ^{1}H-NMR and HPLC, and *S,S′*-(thio*bis*(4,1-phenylene)) dimethanethioate was used without further purification. Yield: 91% (2.79 g), white solid, Mp. 70-72 °C, R_f 0.58 (20% EtOAc-80% cyclohexane).

^{1}H-NMR (200 MHz, DMSO-d_6, δ): 10.38 (s, 2H), 7.44-7.59 (m, 8H) ppm; ^{13}C-NMR (50 MHz, DMSO-d_6, δ): 190.4 (2 SC=O), 136.4 (2 C), 134.9 (2 CH), 131.6 (2 CH), 125.3 (2 C) ppm; FTIR-ATR: ν = 3335.5, 3059.6, 2861.4, 1699.5, 1684.0, 1575.2, 1474.2, 1450.8, 1335.0, 1096.1, 1079.6, 1009.6, 960.2, 943.7, 910.8, 816.0, 774.8, 741.9, 733.6, 696.6 cm^{-1}

2. Kinetic studies by ^{1}H-NMR

The photoreaction of *S*-phenyl thioformate (**1b**) was investigated by ^{1}H-NMR spectroscopy (48.2 mM, CD_3CN, quartz NMR tube, 200 MHz). **Figure S1** shows in detail the ^{1}H-NMR spectra of the sample *S*-phenyl thioformate (**1b**) before irradiation (blank) and after several irradiation doses with UV light (254 nm, 5.08 mWcm^{-2}). During irradiation, the sample in the quartz NMR tube was stirred at 1000 rpm with a vortex. The distance between the NMR tube and the end of the light guide was 3 cm.

Before irradiation (blank), the singlet corresponding to the H of the thioformate (HCOS-) was found at 10.24 ppm. After one hour of overall irradiation, the formyl signal decreased from 1 to 0.06 H giving a conversion of 94%.

The products of the photoreaction were investigated by HPLC (UV detection at 254 nm; **Figure S2**).

The photoreaction of *S,S'*-(thio*bis*(4,1-phenylene)) dimethanethioate (2b) was investigated by ^{1}H-NMR spectroscopy (7.72 mM, DMSO-d_6, borosilicate glass NMR tube, 200 MHz). The sample was irradiated at different irradiation doses with visible light (405 nm, 1.55 W cm^{-2}). During irradiation, the sample in the borosilicate glass NMR tube was stirred with a vortex (1000-2000 rpm). The distance between the NMR tube and the end of the light guide was 2 cm.

For *S,S'*-(thio*bis*(4,1-phenylene)) dimethanethioate, a conversion of 42% was found (see **Figure S3**).

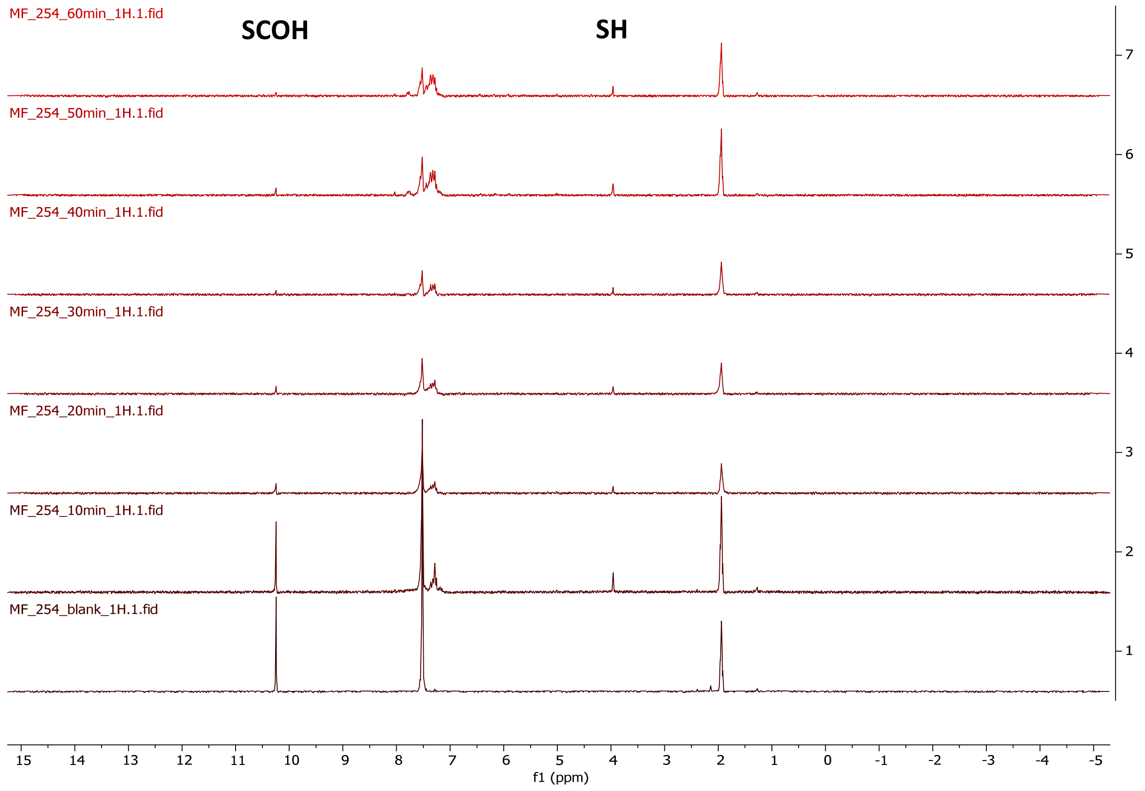

Figure S1: Spectra of *S*-phenyl thioformate (**1b**) at different irradiation doses with monochromatic UV light (254 nm).

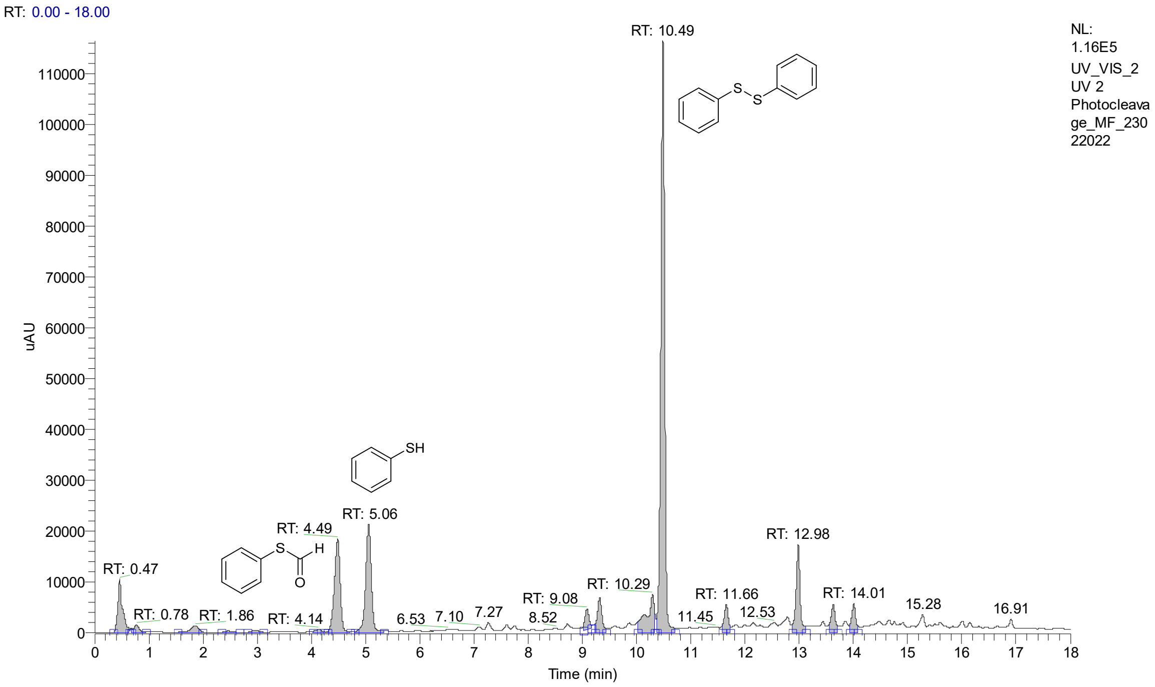

PEAK LIST

Photocleavage_MF_23022022.RAW

RT: 0.00 - 18.00

Number of detected peaks: 15

Apex RT	Start RT	End RT	Area	%Area	Height	%Height
0.47	0.36	0.63	65185.762	5.25	10616.830	4.83
0.78	0.70	0.85	10357.668	0.83	1651.390	0.75
1.86	1.61	1.99	17290.260	1.39	1451.706	0.66
2.96	2.82	3.09	6982.288	0.56	627.370	0.29
4.14	4.01	4.21	6282.027	0.51	742.665	0.34
4.49	4.27	4.62	142297.308	11.46	18452.720	8.40
5.06	4.84	5.25	155466.512	12.53	21361.660	9.73
9.08	8.99	9.17	30394.682	2.45	4886.530	2.22
9.32	9.21	9.41	44292.681	3.57	6997.520	3.19
10.30	9.91	10.35	84358.096	6.80	7580.623	3.45
10.49	10.37	10.60	535053.215	43.11	114279.291	52.03
11.66	11.61	11.77	25255.940	2.03	5233.933	2.38
12.98	12.91	13.11	77267.280	6.22	16466.147	7.50
13.63	13.55	13.68	22669.556	1.83	4970.296	2.26
14.01	13.92	14.08	18089.367	1.46	4321.712	1.97

Figure S2: HPCL chromatogram of photocleavage reaction of *S*-phenyl thioformate (**1b**).

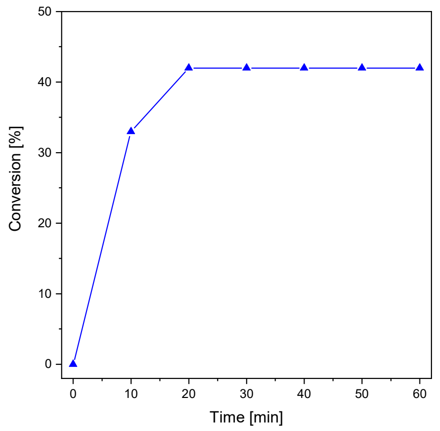

Figure S3: Conversion of *S,S′*-(thio*bis*(4,1-phenylene)) dimethanethioate (**2b**) under visible light (405 nm, 1.55 W cm^{-2}).

3. UV-vis absorption behaviour

The UV-Vis absorption behaviour of the investigated compounds was measured at different concentrations in acetonitrile and in DMSO.

Firstly, a comparison of the maximum absorption of *S*-phenyl thioformate (**1b**), thiophenol and diphenyl disulfide was carried out at 0.001 M in acetonitrile. As shown in the **Figure S4**, thiophenol presents the higher absorption, followed by *S*-phenyl thioformate (**1b**) and diphenyl disulfide.

Then, the absorption of 4,4′-thiol*bis*benzenethiol (**2a**) and *S,S′*-(thio*bis*(4,1-phenylene)) dimethanethioate (**2b**) was studied in detail at different concentrations in DMSO **Figure S5**, found it again that the absorption of the thiol derivative (> 350 nm) is higher than the formyl derivative (< 350 nm).

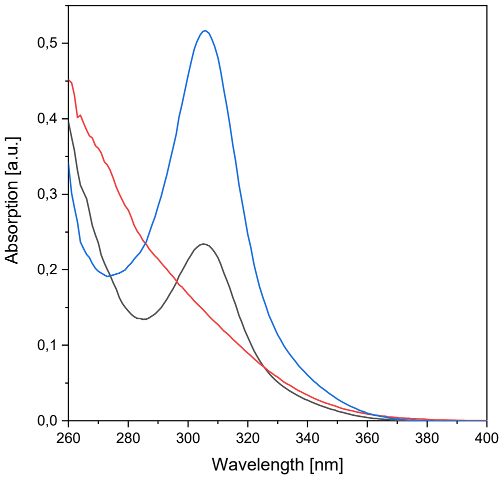

Figure S4: UV spectra of *S*-phenyl thioformate (black line), thiophenol (blue line) and diphenyl disulfide (red line).

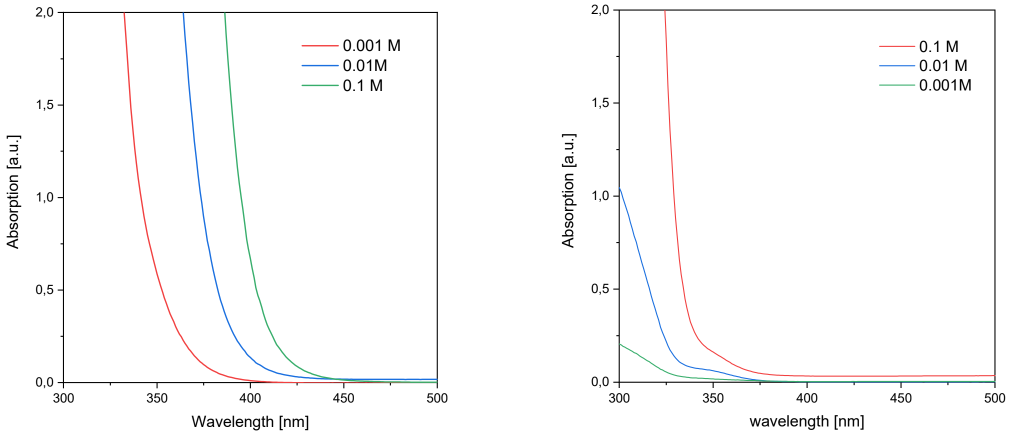

Figure S5: UV-Vis spectra of **2a** (left) and **2b** (right)

4. Further photodecarbonylation studies

4.1. Cabon monoxide (CO) detection by GC-MS

The CO formation was proved by GC-MS, measuring the contained gas in a closed vial, after irradiating *S*-phenyl thioformate (0.1 mL) under Ar atmosphere (**Figure S6**). The sample was irradiated with an OmniCure® lamp series 1000 (100 watt Mercury Arc.) for 1 min at 1 cm of distance.

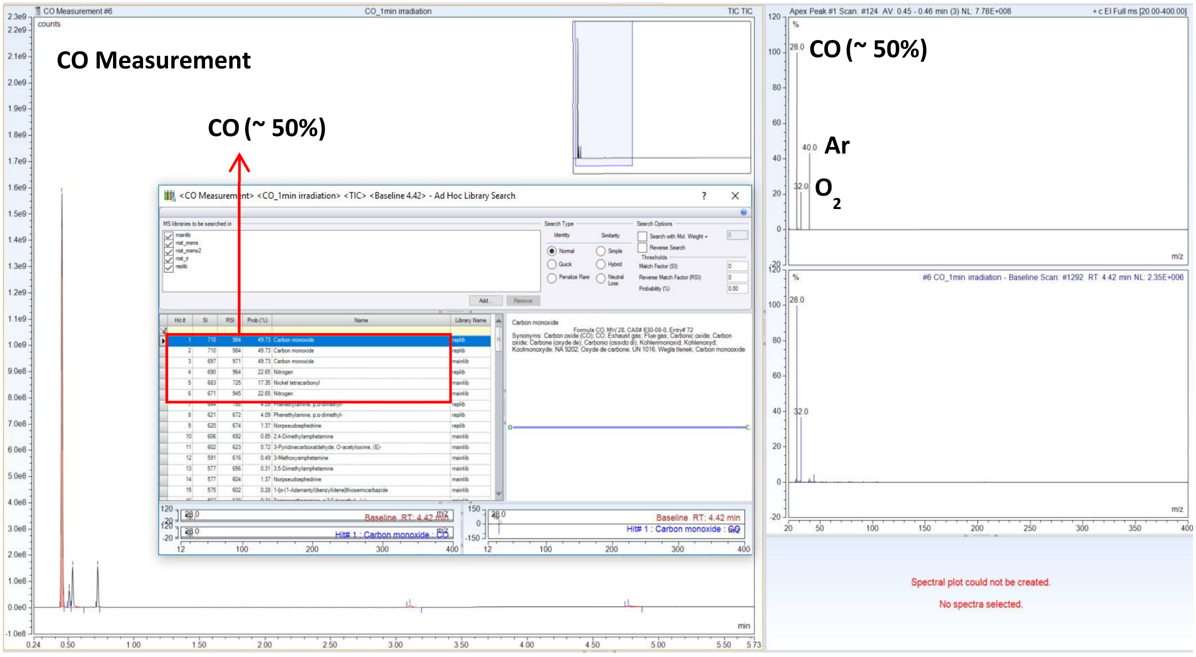

Figure S6: Chromatogram and MS spectra of photodecarbonylation reaction (photocleavage of S-thioformate with generation of carbon monoxide).

To corroborate this result, measurements of two blanks (vial filled with air and argon) were conducted as it shown in **Figure S7**.

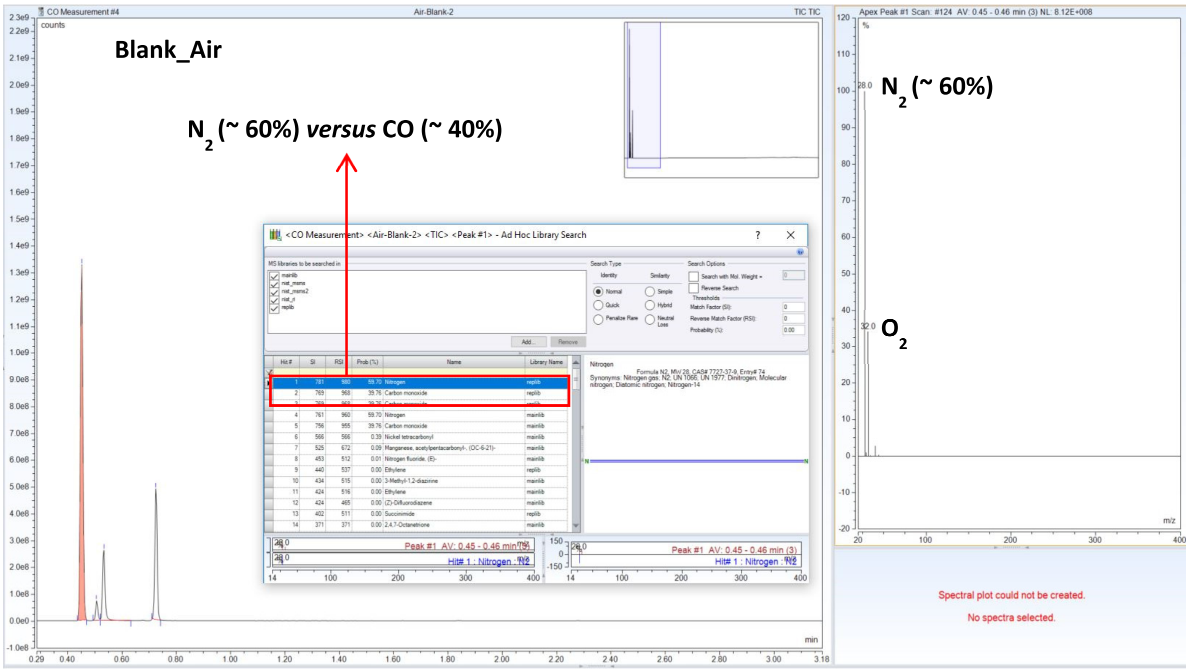

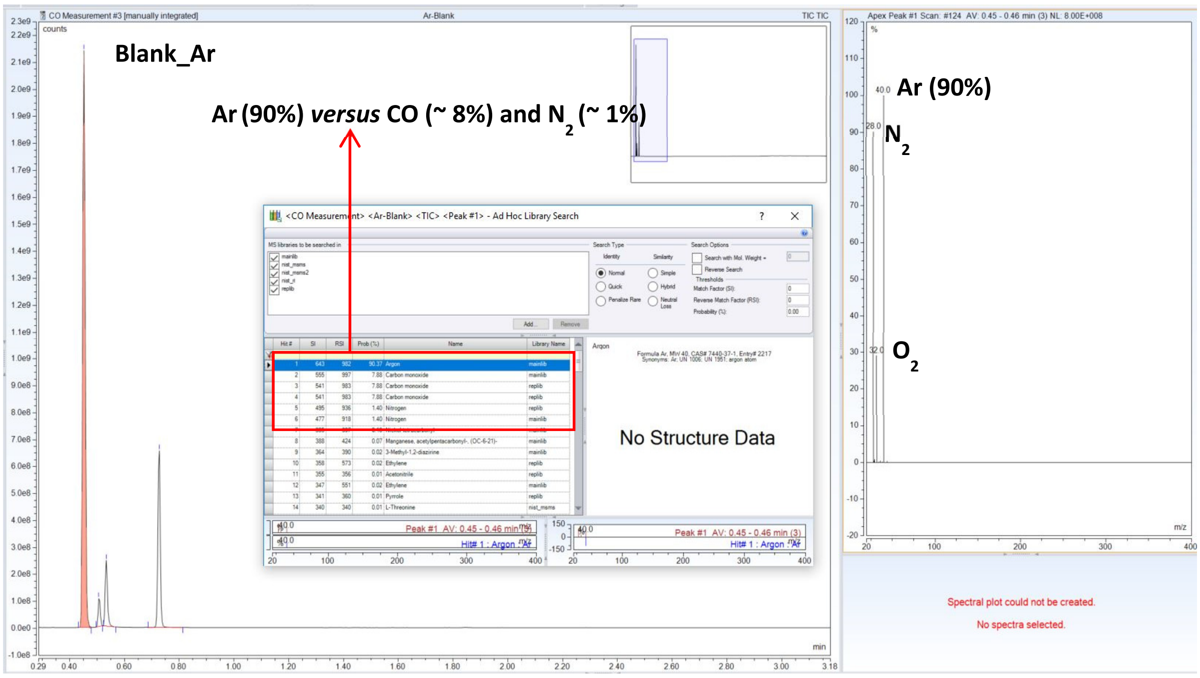

Figure S7: Chromatograms and MS spectra of blanks samples. The upper figure corresponds to air measurement and the lower figure to argon measurement.

4.2. Detection of formed radicals by spin-trapping electron paramagnetic resonance (ST-EPR) spectroscopy.

The generation of thiyl (PhS·), formyl (CHO·) and hydrogen (H·) radicals were confirmed by comparison of the hyperfine coupling constants (a_N and a_H) obtained in the simulation with reference data shown in Table S1. [1–3] Figure S8 displays the signal assignations in the spectrum.

Table S1. Simulation parameters of the EPR spectra displayed in the paper, together with published reference data.

Radical	Relative Intensity (%)	a_N (mT)	a_H (mT)	Reference
**PhS•**	51	1.36<br>1.36	1.47<br>1.45	This work<br>[31]
**CHO•**	39	1.50<br>1.58	2.06<br>2.12	This work<br>[32,33]
**H•**	10	1.66<br>1.61	2.07 (2 H)<br>2.25 (2 H)	This work<br>[33]

The simulation of the three radical species shows a good agreement with previously published data. Especially the hyperfine couplings for the thiyl radical **PhS•** show an essentially prefect fit. The other two radicals show small deviations. This can be explained by the different solvent used in our experiment.

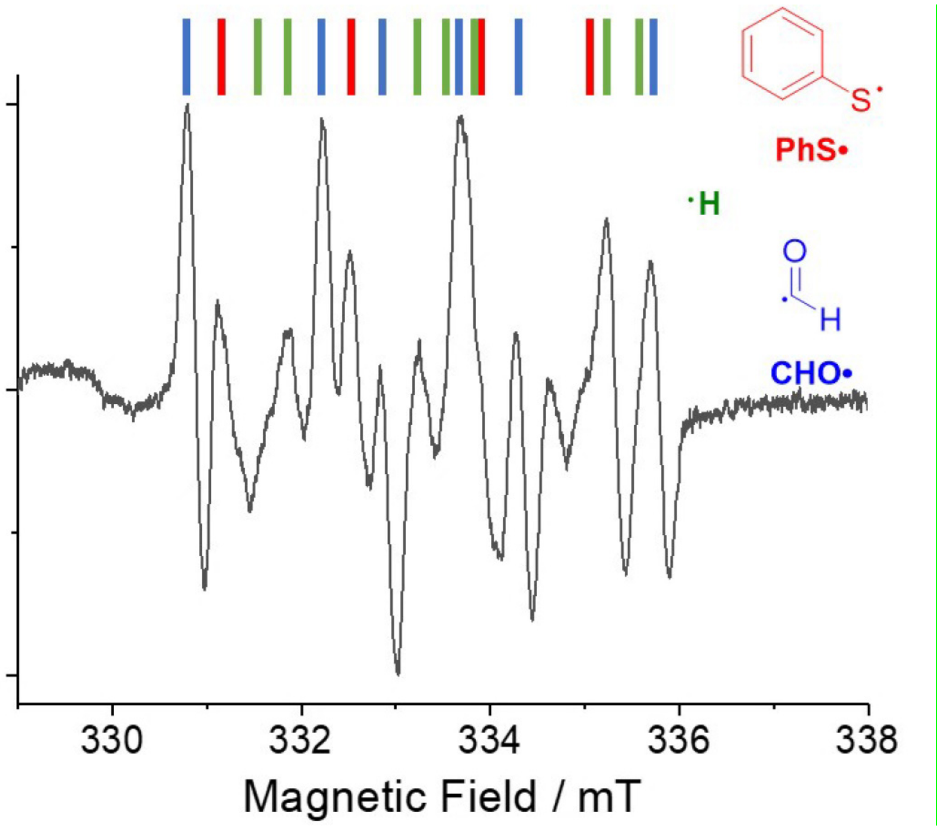

Figure S8: Spectrum signal assignment.

5. Formulations

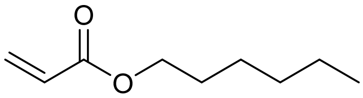

Hexyl acrylate formulations: these formulations were prepared using 1 mM of photoinitiator (PI) [**1a**, **1b** and **the corresponding disulfide**] to calculate the double bond conversion (DB conversion) by IR.

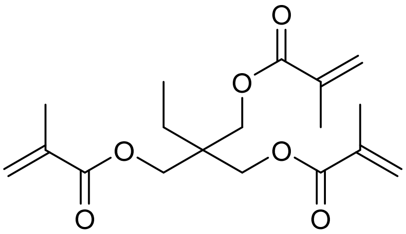

Trimethylolpropan-trimethacrylat (TMPTMA) formulations: these formulations were prepared using 1 mM of PI [**2a** (bifunctional thiol) or **2b** (bifunctional *S*-thioformate)]to calculate the DB conversion by IR. For printing, 0.01 wt% Sudan II G (light absorber) and 0.5 wt% pyrogallol (stabilizer) were added to the formulations.

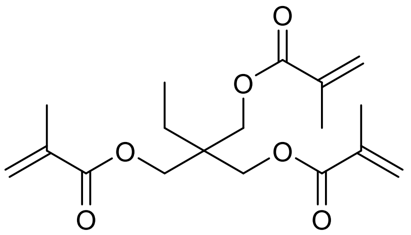

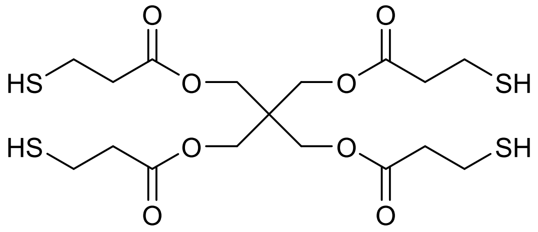

Trimethylolpropan-trimethacrylat (TMPTMA) and (PETMP) formulations: these formulations were prepared using 70 mol% TMPTMA [ene] and 30 mol% PETMP [SH] containing 0.5 wt% propyl gallate (radical scavenger) and 1 mM PI [**2a** (bifunctional thiol) or **2b** (bifunctional *S*-thioformate)], both to calculate the DB conversation by IR and to show their stability by rheological measurements.

6. Appendix of ^{1}H- and ^{13}C-NMR spectra

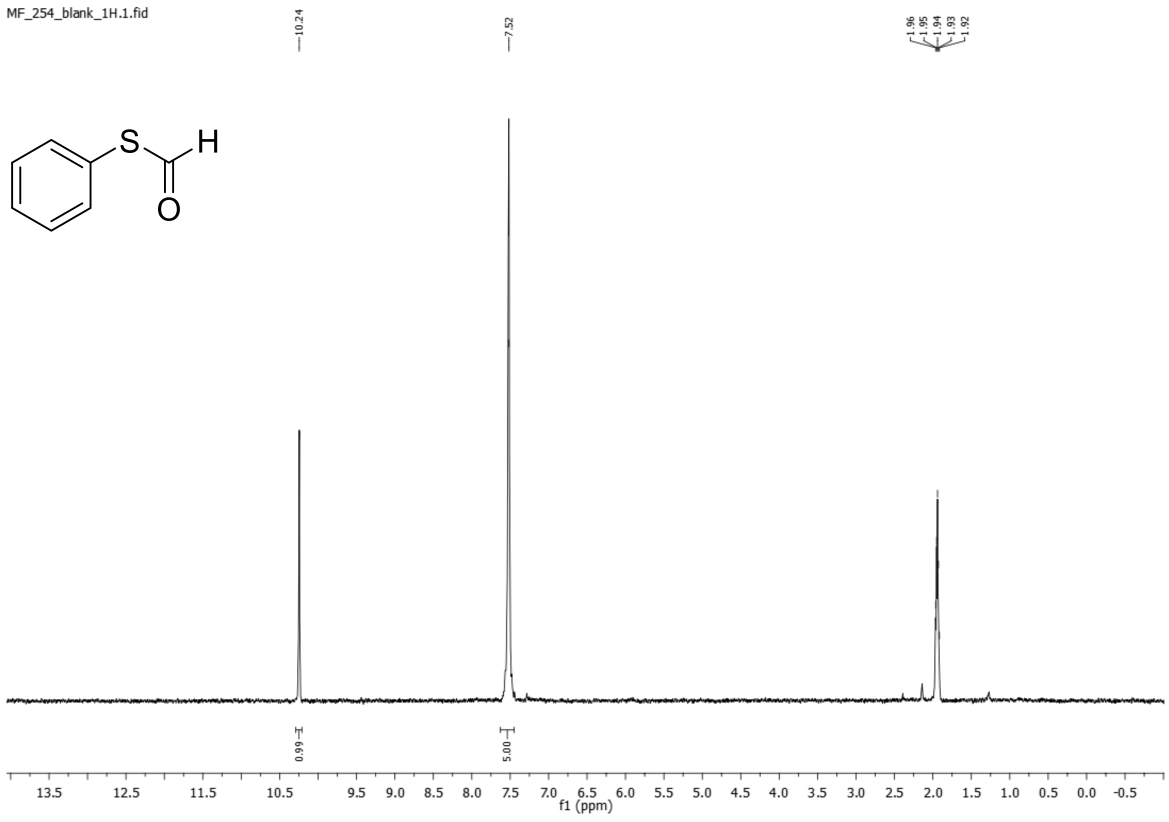

Figure S9: ^{1}H-NMR (200 MHz, CD_3CN) of *S*-phenyl thioformate (**1b**)

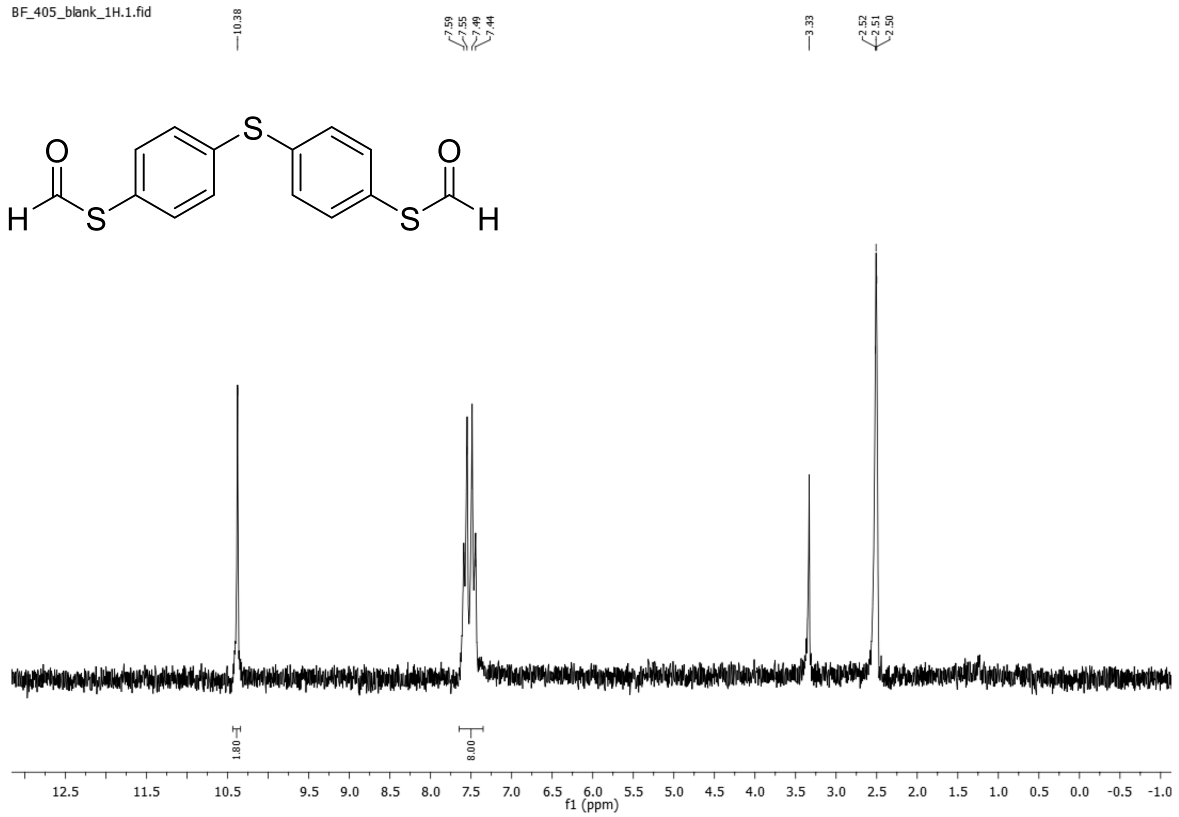

Figure S10: ^{1}H-NMR (200 MHz, DMSO-d_6) of *S,S'*-(thio*bis*(4,1-phenylene)) dimethanethioate (**2b**)

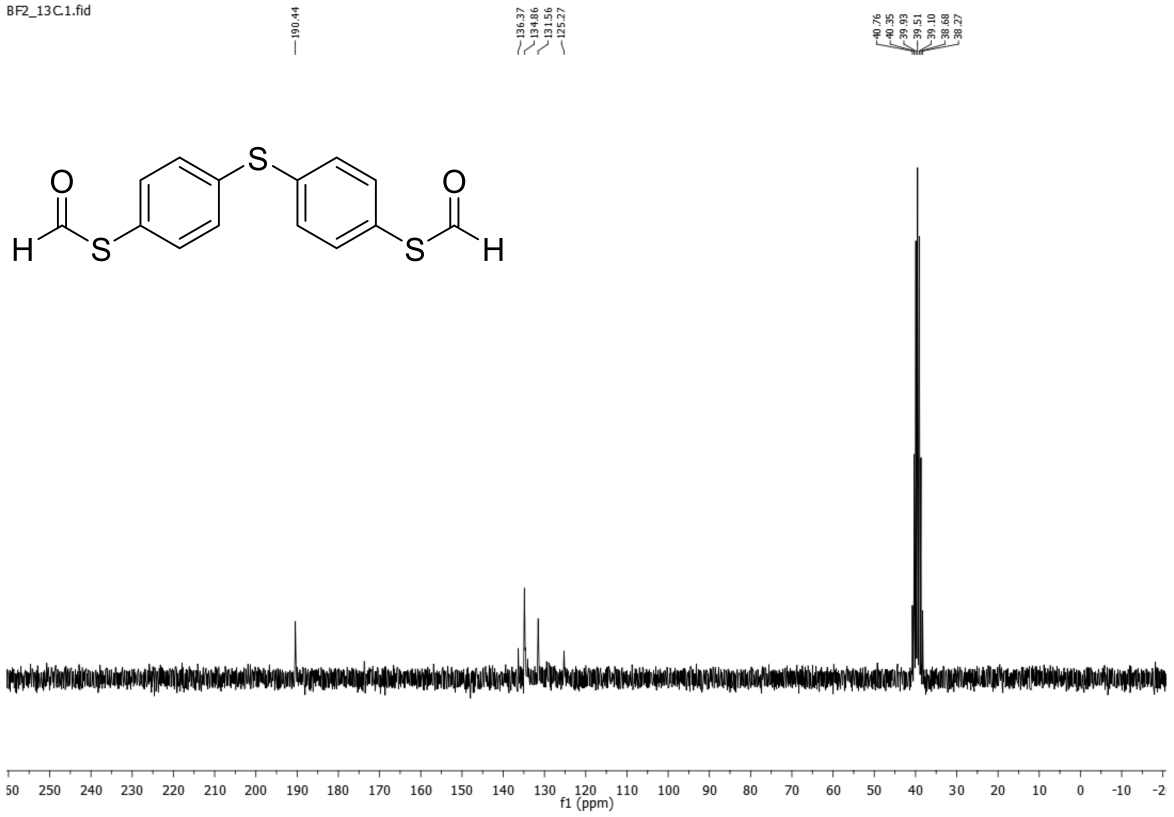

Figure S11: ^{13}C-NMR (50 MHz, DMSO-d_6) of *S,S'*-(thio*bis*(4,1-phenylene)) dimethanethioate (**2b**)
